# Supplementary material for: Parylene-Sealed Perovskite Nanocrystals Down-Shifting Layer for Luminescent Spectral Matching in Thin Film Photovoltaics
Source: Nanomaterials (Basel). 2023 Jan 3;13(1):210. doi: 10.3390/nano13010210 (PMC9823874; doi:10.3390/nano13010210)
Supplement: Supplementary file 1 [file nanomaterials-13-00210-s001.zip › nanomaterials-2116466-supplementary.pdf]

# Supplementary Materials

Ana Pinheiro, Andreia Ruivo, João Rocha, Marta Ferro, Joana Vaz Pinto, Jonas Deuermeier, Tiago Mateus, Ana Santa, Manuel J. Mendes, Rodrigo Martins, Sandra Gago, César A.T. Laia, Hugo Águas

**Table S1**: Description of the deposition parameters, from which the hydrogen dilution (HD) and the ratio of doping gas (R), for the production of nc-Si:H PV cells by PECVD.

| n-Si:H cell layer | Power density (mW/cm²) | Frequency (MHz) | Electrode distance (mm) | Temperature (°C) | Pressure (Torr) | HD (%) | R(%) | Deposition time (s) |
|---|---|---|---|---|---|---|---|---|
| n | 69.44 | 13.56 | 10 | 180 | 2 | 96.7 | 1 | 16'20" |
| n (a) | 20.83 | 13.56 | 16.5 | 170 | 1 | 66.67 | 3 | 56" |
| seed layer 3 | 138.89 | 75 | 23-13 | 170 | 1 | 98.4-98.5 | 0 | 10' |
| i | 138.89 | 75 | 13 | 170 | 1 | 97.3 | 0 | 1h53'39" |
| p | 90.28 | 13.56 | 10 | 170 | 2.4 | 99.29 | 0.56 | 4'54" |

**Table S2**: Description of the deposition parameters for the production of $CsPbBr_3$ NCs thin films, from the colloidal perovskite, on nc-Si:H PV cells, via spin-coating.

| Glass sample with deposited nc-Si:H PV cells (nr. of depositions; concentration NCs) | NCs to 10% (weight) polyethylene-hexane solution ratio | Spin-coater parameters | Thermal annealing |
|---|---|---|---|
| (1 deposition; pellet re-dispersed in 2.5 mL of hexane) | 1:2, followed by an thermal anneal | Time: 30 s<br>Velocity: 1500 rpm<br>Acceleration: 500 rpm/s | 50 ºC during 5 min |

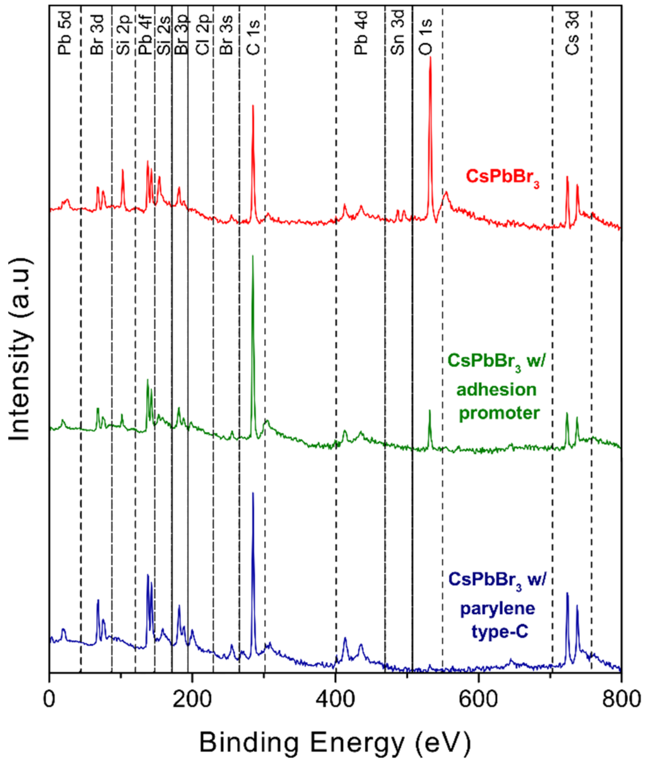

**Figure S1:** XPS spectra for the different elements present in the $CsPbBr_3$ NCs thin film, the $CsPbBr_3$ NCs thin film with the incorporation of the adhesion promotor and the $CsPbBr_3$ NCs-parylene type C thin film.

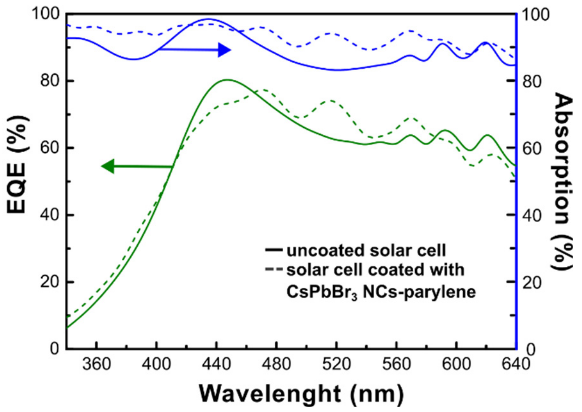


**Figure S2:** EQE (green lines) and absorption (blue lines) of the solar cell of replica i before (solid line) and after (dashed line) the incorporation of the CsPbBr3 NCs-parylene type C thin film.

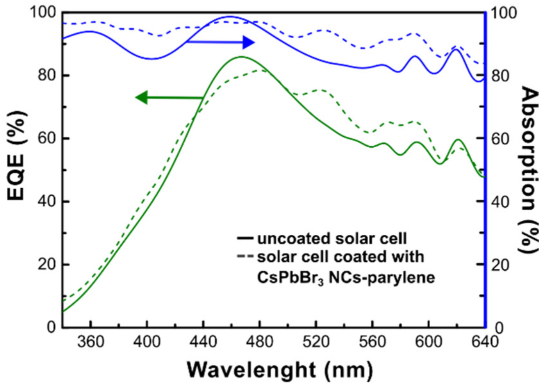


**Figure S3:** EQE (green lines) and absorption (blue lines) of the solar cell of replica iii before (solid line) and after (dashed line) the incorporation of the CsPbBr3 NCs-parylene type C thin film.

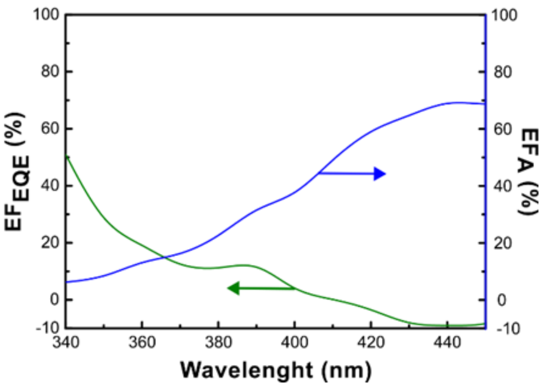


**Figure S4:** Enhancement factor for the EQE and absorption of the i solar cell replica, calculated from the relative percentage differences between the uncoated PV cell and the coated PV cells with the CsPbBr3 NCs-parylene type C thin film.

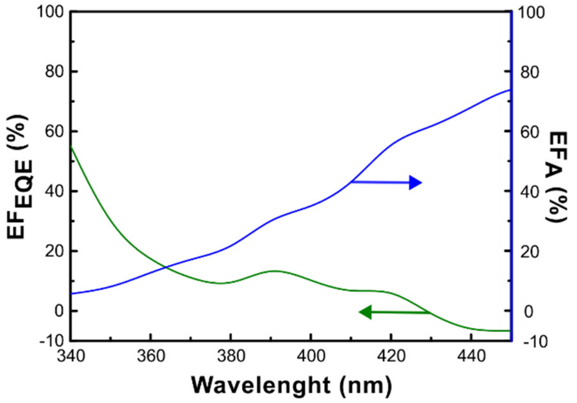


**Figure S5:** Enhancement factor for the EQE and absorption of the ii solar cell replica, calculated from the relative percentage differences between the uncoated PV cell and the coated PV cells with the CsPbBr3 NCs-parylene type C thin film.

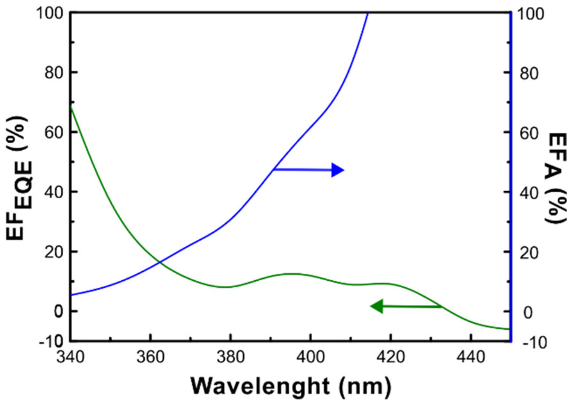


**Figure S6:** Enhancement factor for the EQE and absorption of the iii solar cell replica, calculated from the relative percentage differences between the uncoated PV cell and the coated PV cells with the CsPbBr3 NCs-parylene type C thin film.

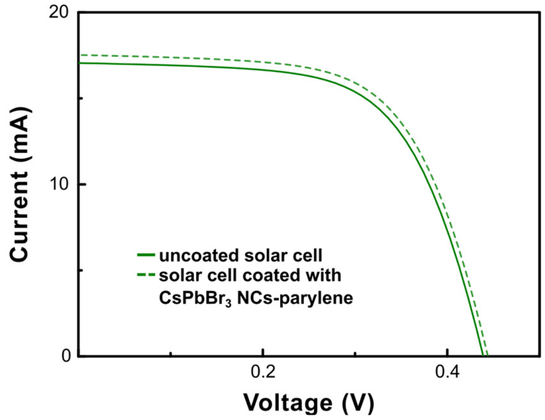


**Figure S7:** I-V curve of replica ii before (solid green line) and after (dashed green line) the incorporation of the CsPbBr3 NCs-parylene type C thin film.

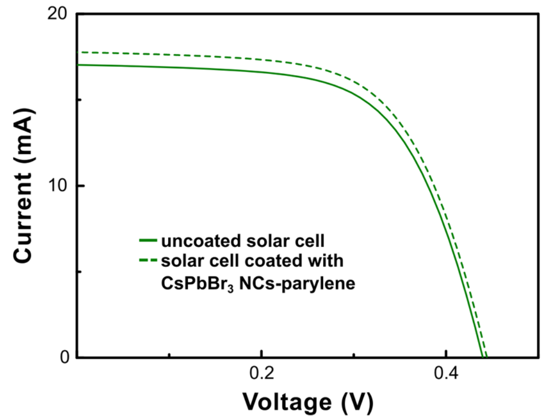


**Figure S8:** I-V curve of replica iii before (solid green line) and after (dashed green line) the incorporation of the CsPbBr3 NCs-parylene type C thin film.

**Table S3**: Measured photovoltaic cell parameters for replicas i, ii, and iii, when uncoated and coated with the $CsPbBr_3$ NCs-parylene type C thin film: open-circuit voltage, short-circuit current, short-circuit current density, maximum current, maximum voltage, maximum power, fill factor and efficiency.

| PV cell replica | Voc (V) | Isc (mA) | Jsc (mA/cm²) | Imax (mA) | Vmax (V) | Pmax (mW) | Fill Factor (%) | Efficiency (%) |
|---|---|---|---|---|---|---|---|---|
| i uncoated | 0.44 | 3.20 | 17.14 | 2.72 | 0.32 | 0.87 | 61.63 | 4.65 |
| i coated | 0.45 | 3.31 | 17.78 | 2.82 | 0.32 | 0.91 | 61.68 | 4.89 |
| ii uncoated | 0.44 | 3.18 | 17.06 | 2.72 | 0.32 | 0.87 | 62.67 | 4.69 |
| ii coated | 0.44 | 3.28 | 17.60 | 2.80 | 0.33 | 0.91 | 62.75 | 4.91 |
| iii uncoated | 0.44 | 3.18 | 17.04 | 2.71 | 0.32 | 0.87 | 62.49 | 4.68 |
| iii coated | 0.43 | 3.25 | 17.45 | 2.83 | 0.33 | 0.94 | 66.18 | 5.02 |

Method details

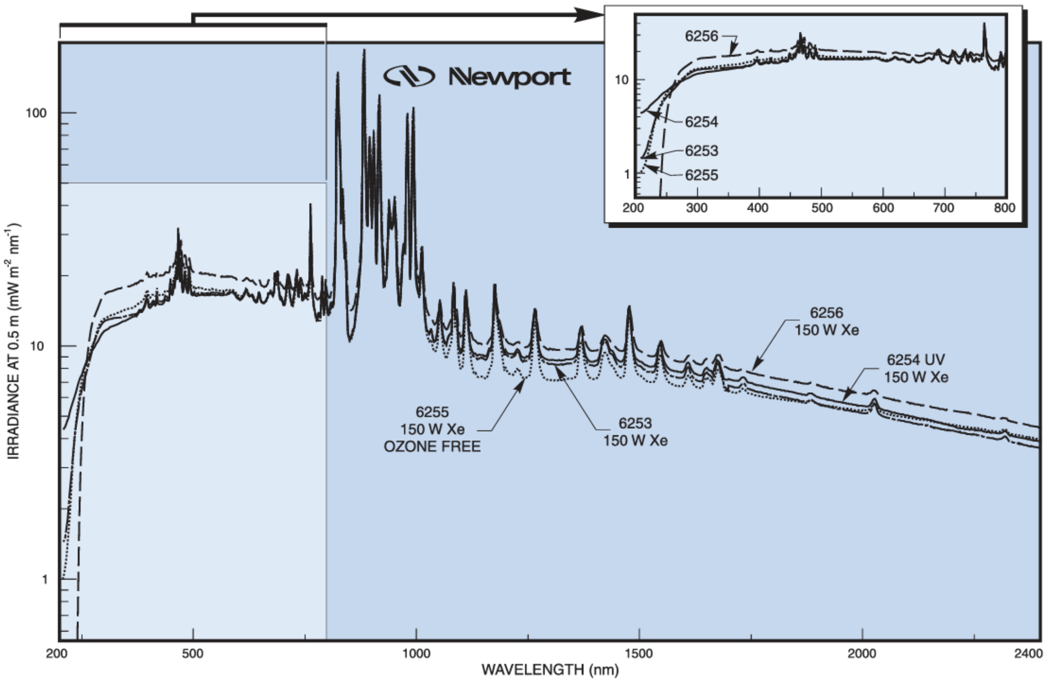


This is the spectral irradiance profile from the used irradiation source for the spectral response measurements (using a Newport-Oriel ® 6255, 150W, Ozone Free Xe ARC Lamp). This information is provided by the fabricant and can be consulted in the following website: https://www.newport.com/p/6255
